# The longitudinal interplay among L2 writing self-efficacy, self-regulated writing strategies, and writing performance: a three-wave cross-lagged panel study of Chinese EFL learners

**DOI:** 10.3389/fpsyg.2026.1972788

**Published:** 2026-10-16

**Authors:** Dongliang Ding, Ahmad Muhyiddin Bin Yusof

**Affiliations:** 1 School of Foreign Languages, Nanyang Normal University, Nanyang, China; 2 Academy of Language Studies, Universiti Teknologi MARA, Shah Alam, Malaysia

**Keywords:** cross-lagged panel model, EFL learners, L2 writing self-efficacy, longitudinal study, self-regulated writing strategies, writing performance

## Abstract

Writing in a second language (L2) is a complex cognitive activity that demands sustained self-regulation and a strong sense of personal capability. Although cross-sectional studies have linked writing self-efficacy, self-regulated writing strategies, and writing performance, the directional and reciprocal nature of these relationships remains insufficiently understood. This three-wave longitudinal study examined the temporal dynamics among L2 writing self-efficacy (L2WSE), self-regulated writing strategies (SRWS), and writing performance (WP) over one academic semester. A total of 368 Chinese EFL learners (Mage = 19.2 years, SD = 1.1; 62.2% female) from a public university in central China completed self-report measures of L2WSE and SRWS at three time points (Week 1, Week 8, and Week 16) and produced a timed writing task at each wave. Confirmatory factor analyses supported the three-factor measurement model, and tests of measurement invariance confirmed configural, metric, and scalar invariance across waves. A cross-lagged panel model (CLPM) revealed that (a) L2WSE prospectively predicted SRWS from T1 to T2 (*β* = 0.21, *p* < 0.001) and from T2 to T3 (*β* = 0.19, *p* < 0.01), (b) SRWS prospectively predicted WP from T1 to T2 (*β* = 0.16, *p* < 0.01) and from T2 to T3 (*β* = 0.18, *p* < 0.01), and (c) WP prospectively predicted L2WSE from T1 to T2 (*β* = 0.17, *p* < 0.01) and from T2 to T3 (*β* = 0.15, *p* < 0.05). A random-intercept cross-lagged panel model (RI-CLPM) sensitivity analysis yielded directionally consistent within-person effects, with slightly attenuated standardized coefficients. The findings suggest a temporally ordered, sequential chain in which self-efficacy beliefs prospectively predict greater strategic engagement, which in turn predicts higher subsequent writing outcomes, while successful writing experiences predict higher subsequent self-efficacy. These model-implied longitudinal associations extend Bandura’s social cognitive theory to L2 writing contexts and highlight the pedagogical importance of nurturing both motivational beliefs and strategic competence in EFL writing instruction, although the observational design precludes causal conclusions.

## 1 Introduction

Writing in a second language (L2) is widely recognized as one of the most demanding skills for learners to acquire, requiring the integration of linguistic knowledge, strategic competence, and motivational resources (Flower and Hayes, 1981; Hayes, 2012). Within self-regulated learning (SRL) research, writing occupies a unique position because it involves recursive cognitive processes that unfold over extended periods and demand sustained self-monitoring (Zimmerman, 2002; Zimmerman and Risemberg, 1997). Central to this self-regulatory endeavor are two constructs that have garnered increasing attention in L2 writing research: writing self-efficacy and self-regulated writing strategies.

The cognitive process model of writing, first articulated by Flower and Hayes (1981) and later revised by Hayes (1996, 2012), provides a foundational framework for understanding the interplay of motivation and cognition in writing. In its original form, the model posited three core processes (planning, translating, and reviewing) operating within a working memory system. The revised version (Hayes, 1996) incorporated motivational components such as goals, beliefs, and attitudes, explicitly acknowledging that writing is not a purely cognitive activity but one intertwined with self-regulatory and motivational processes. Most recently, Hayes (2012) proposed a three-layer architecture comprising control, process, and resource levels, further reinforcing the role of self-regulation in coordinating writing activities. These theoretical developments highlight the need to examine how motivational beliefs and strategic processes interact dynamically over time to shape writing outcomes.

Writing self-efficacy, defined as learners’ beliefs in their capability to produce written text, originates from Bandura’s (1986, 1997) social cognitive theory. According to this perspective, self-efficacy is not merely a passive correlate of performance but an active motivational determinant that shapes the effort, persistence, and strategic approaches learners employ (Bandura, 2012). In the domain of L2 writing, self-efficacy beliefs have been shown to predict writing performance, strategy use, and motivational outcomes such as engagement and anxiety (Pajares, 2003; Pajares and Valiante, 2006; Zhou et al., 2022). Bruning et al. (2013) further advanced the construct by proposing a multidimensional model of writing self-efficacy encompassing ideation (generating content), conventions (mechanical and grammatical correctness), and self-regulation (managing the writing process). This multidimensional view has been validated in L2 contexts (Sun et al., 2022), supporting its applicability across language learning settings.

Self-regulated writing strategies (SRWS) refer to the deliberate processes that learners deploy to plan, monitor, and revise their writing (Zimmerman and Risemberg, 1997; Graham and Perin, 2007). Drawing on Zimmerman’s (2000) three-phase cyclical model of SRL—forethought (goal setting and task analysis), performance (self-instruction and self-monitoring), and self-reflection (self-evaluation and adaptive reactions)—SRWS encompass goal planning, idea generation, text organization, monitoring, and revision. Teng et al. (2022) developed a self-regulatory writing strategy questionnaire specifically for EFL learners, demonstrating its predictive validity for writing performance. Their work established a direct link between strategic engagement and writing outcomes in L2 settings.

A substantial body of empirical evidence has linked writing self-efficacy, strategy use, and writing performance. Zimmerman and Bandura (1994) demonstrated that self-efficacy and self-regulation jointly predicted writing achievement in a two-wave longitudinal study of college students. Pajares (2003) further showed that writing self-efficacy was a stronger predictor of writing performance than past performance itself, particularly among secondary school students. In a meta-analysis of writing instruction, Graham and Perin (2007) identified strategy instruction as one of the most effective interventions for improving adolescent writing. More recently, Bruning et al. (2013) demonstrated that their multidimensional writing self-efficacy scale predicted writing performance, and Sun et al. (2022) validated the scale with Chinese EFL learners, confirming its cross-cultural applicability. Zhou et al. (2022) further extended this line by showing that L2 writing self-efficacy, self-regulated strategies, and engagement were interrelated among Chinese high school students. Empirical work published in the past 3 years has consolidated these links in Chinese EFL contexts. Teng and Zhan (2023) showed, through structural equation modeling with Chinese undergraduates, that writing self-efficacy and self-regulated writing strategies jointly mediated the effects of task complexity on academic writing performance. Bai and Wang (2023) demonstrated that self-regulated reading-to-write strategies were closely tied to motivation and writing competence among EFL learners, and Chen et al. (2026) identified distinct latent profiles of self-regulated learning strategies and self-efficacy that were differentially associated with writing achievement. Teng (2024) further showed that motivational beliefs and self-efficacy predicted multiple dimensions of self-regulated learning strategies in EFL writing among Chinese undergraduates, and Zhu and Sun (2025) reported that both writing self-efficacy and strategy use varied systematically across writing proficiency levels among Chinese English majors. Intervention evidence is also accumulating: Rahimi and Fathi (2022) found that wiki-mediated collaborative writing instruction improved EFL students’ writing performance, writing self-regulation, and writing self-efficacy over the course of one term.

Despite the accumulating body of evidence, several gaps warrant attention. First, most prior studies have relied on cross-sectional designs, which preclude causal inferences about the direction of effects among self-efficacy, strategies, and performance (Honicke and Broadbent, 2016). The temporal ordering implied by social cognitive theory—that self-efficacy drives strategy use, which in turn shapes performance—has rarely been tested longitudinally in L2 writing. Second, the possibility of reciprocal effects, wherein performance outcomes feedback to influence subsequent self-efficacy (Bandura, 1997), remains underexplored. In social cognitive theory, mastery experiences are considered the most powerful source of self-efficacy information, implying that successful writing performance should strengthen subsequent self-efficacy beliefs. Longitudinal evidence from academic domains more broadly (Honicke and Broadbent, 2016) suggests reciprocal effects between self-efficacy and performance, yet this feedback dynamic has not been tested in L2 writing with multi-wave data. Third, few studies have simultaneously modeled all three constructs in a unified longitudinal framework. Teng et al. (2024) examined motivation and strategy use longitudinally but did not include writing performance as an outcome; Dong (2025) tested a three-wave mediation model linking motivational regulation strategies to writing proficiency but did not model writing self-efficacy; and cross-sectional studies that modeled motivational and strategic constructs together (e.g., Zhou et al., 2022; Teng and Zhan, 2023) preclude directional inferences. Consequently, it remains unclear whether self-efficacy influences performance directly, indirectly through strategies, or both.

The cross-lagged panel model (CLPM) is well-suited to address these gaps. By examining autoregressive (stability) and cross-lagged (directional) paths among variables measured at multiple time points, CLPM allows researchers to evaluate temporal precedence and reciprocal effects (Selig and Preacher, 2009). However, traditional CLPM has been critiqued for confounding between-person and within-person variance (Hamaker et al., 2015). The random-intercept CLPM (RI-CLPM) addresses this limitation by separating trait-like between-person differences from within-person fluctuations (Usami et al., 2019). The present study employs both approaches to provide a rigorous test of directional effects.

Recently, Teng et al. (2024) conducted a longitudinal study examining L2 learners’ motivations and strategic behavior in writing instruction, demonstrating the value of temporal designs in uncovering the dynamic interplay between motivation and strategy use. However, their focus was on motivation broadly rather than on the specific construct of writing self-efficacy, and they did not include writing performance as an outcome in the cross-lagged framework. The present study extends this line of research by simultaneously modeling L2 writing self-efficacy (L2WSE), self-regulated writing strategies (SRWS), and writing performance (WP) across three waves.

The present study employed a three-wave longitudinal design with approximately eight-week intervals, chosen to balance temporal sensitivity with participant retention and to align with instructional cycles in a Chinese university semester. Three measurement occasions allow the estimation of both autoregressive stability and cross-lagged directional paths across two transitions, and provide sufficient temporal lags for the hypothesized motivational–strategic–performance dynamics to unfold.

This study aims to answer three research questions: (1) Does L2WSE prospectively predict SRWS and WP over time? (2) Does SRWS prospectively predict WP over time? (3) Are there reciprocal effects, such that WP prospectively predicts L2WSE and SRWS? Drawing on social cognitive theory (Bandura, 1997) and the SRL framework (Zimmerman, 2000), we hypothesize that L2WSE prospectively predicts SRWS (Hypothesis 1), that SRWS prospectively predicts WP (Hypothesis 2), and that WP prospectively predicts L2WSE, reflecting a feedback loop (Hypothesis 3) (Figure 1).

**Figure 1:**
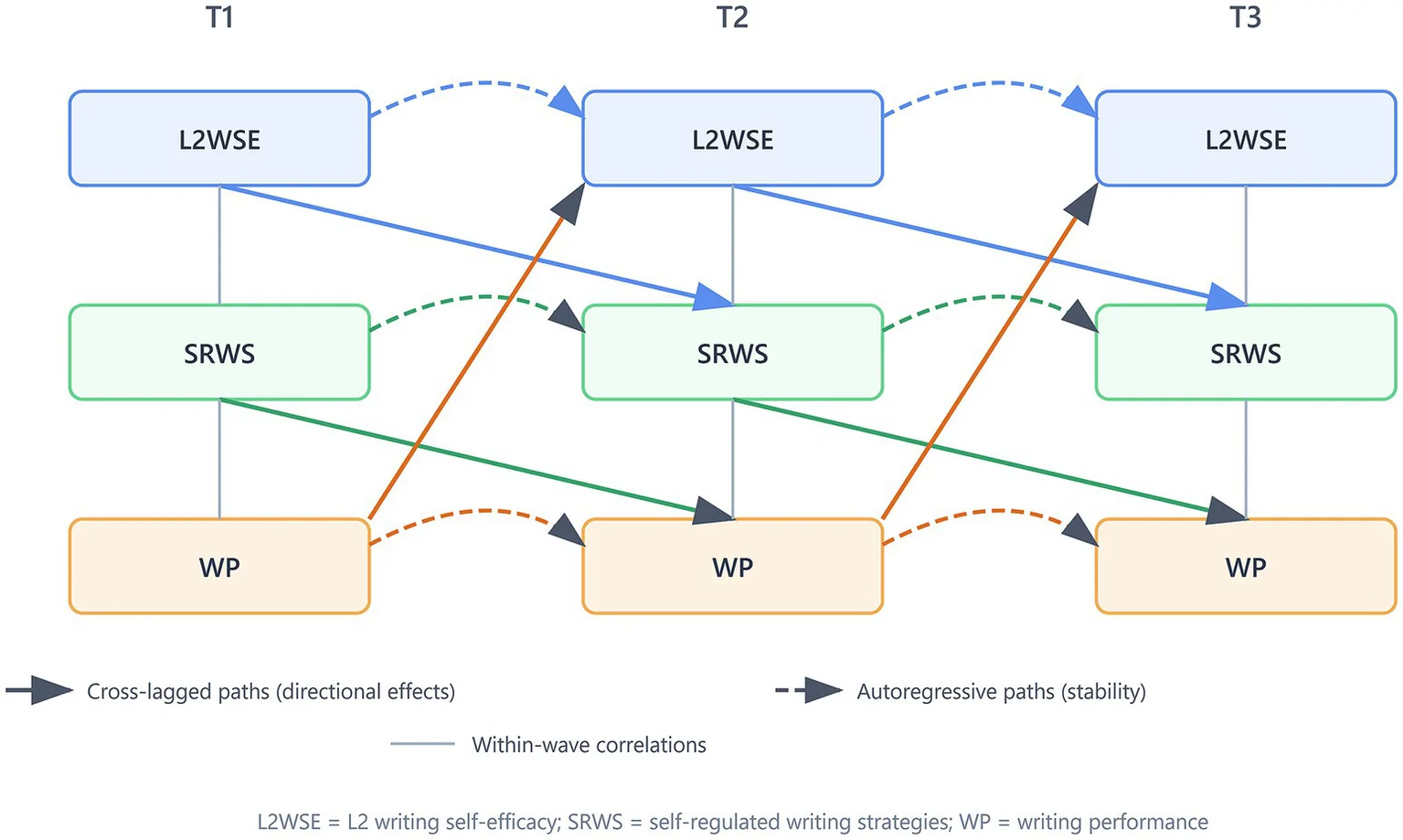
Hypothetical cross-lagged panel model depicting autoregressive stability paths (dashed arrows), cross-lagged directional paths (solid arrows), and within-wave correlations (gray vertical lines) among L2 writing self-efficacy (L2WSE), self-regulated writing strategies (SRWS), and writing performance (WP) across three measurement waves (T1, T2, T3).

## 2 Materials and methods

### 2.1 Participants

Participants were 368 undergraduate EFL learners recruited from a public university in central China (Nanyang Normal University, Henan Province). The sample comprised 229 females (62.2%) and 139 males (37.8%), with ages ranging from 17 to 23 years (*M* = 19.2, SD = 1.1). They were native Chinese speakers enrolled in English language courses and had been learning English for at least 8 years (*M* = 10.3, SD = 2.1). The majority were in their first (38.6%) or second (35.1%) year of study, with the remainder in their third (26.3%) year. English was their primary foreign language. Written informed consent was obtained from all participants prior to data collection. For participants under 18 years of age, written informed consent was additionally obtained from a parent or legal guardian, and written assent was obtained from the student.

*A priori* power analysis was conducted to determine the minimum sample size required for the planned cross-lagged panel analysis. Based on medium effect sizes (*f^2^* = 0.15) expected from previous longitudinal studies of self-efficacy and self-regulation (Honicke and Broadbent, 2016; Teng et al., 2024), an alpha level of 0.05, and a desired power of 0.95, the analysis indicated a minimum of 107 participants. However, because structural equation modeling with latent variables typically requires larger samples to ensure stable parameter estimates—with recommendations ranging from 10 to 20 cases per estimated parameter (Kline, 2016)—and because the RI-CLPM sensitivity analysis requires larger samples to estimate random intercepts reliably (Usami et al., 2019), we aimed to recruit at least 400 participants. The final sample of 368 retained participants exceeded the minimum requirement and provided sufficient power for all planned analyses.

### 2.2 Measures

#### 2.2.1 L2 writing self-efficacy

Writing self-efficacy was assessed using the Self-Efficacy for Writing Scale (SEWS) developed by Bruning et al. (2013). The SEWS comprises 15 items distributed across three subscales: Ideation (5 items; e.g., “I can think of good ideas to write about”), Conventions (5 items; e.g., “I can use correct grammar”), and Self-Regulation (5 items; e.g., “I can get back to writing if I get distracted”). Participants rated each item on a 5-point Likert scale ranging from 1 (strongly disagree) to 5 (strongly agree). The SEWS has been validated in L2 contexts with Chinese EFL learners (Sun et al., 2022). In the present study, Cronbach’s alpha values were 0.86, 0.84, and 0.85 for the three subscales at T1, respectively, and composite reliability ranged from 0.82 to 0.88 across waves.

#### 2.2.2 Self-regulated writing strategies

Self-regulated writing strategies were measured using the Self-Regulatory Writing Strategy Questionnaire (SRWSQ) developed by Teng et al. (2022). The questionnaire contains 20 items assessing four dimensions: goal planning (5 items), idea generation (5 items), monitoring (5 items), and revising (5 items). Sample items include “I set specific goals before writing” and “I monitor whether my writing is on track.” Participants responded on a 5-point Likert scale ranging from 1 (never) to 5 (always). The SRWSQ has demonstrated good psychometric properties with EFL learners (Teng et al., 2022). Cronbach’s alpha in this study was 0.88 at T1, 0.89 at T2, and 0.90 at T3.

#### 2.2.3 Writing performance

Writing performance was assessed using a standardized 30-min timed argumentative writing task administered at each wave. To minimize practice effects while maintaining task comparability, three different prompts of equivalent difficulty were used across waves. Prompts were selected from a bank of previously validated argumentative topics and pilot-tested with 30 EFL learners from the same institution; one-way ANOVA confirmed no significant difference in mean essay scores across the three prompts (*F* (2, 27) = 0.42, *p* = 0.66), supporting comparable difficulty (T1: “Should university students be required to take physical education courses?”; T2: “Should social media platforms be regulated by the government?”; T3: “Should homework be abolished in primary schools?”). All prompts required participants to take a position and support it with arguments. Two trained and experienced raters independently scored each essay using an analytic rating scale adapted from the ESL Composition Profile (Jacobs et al., 1981), evaluating content (0–5), organization (0–5), language use (0–5), and mechanics (0–5), yielding a maximum score of 20. The average of the four dimension scores (0–5 metric) was used in all analyses. Raters were blind to the wave of data collection and to participants’ self-efficacy and strategy scores. Inter-rater reliability was established using intra-class correlation coefficients (ICCs), which ranged from 0.84 to 0.89 across the three waves. Discrepancies exceeding one point were resolved through discussion.

### 2.3 Procedure

Data were collected at three time points across one 16-week academic semester: T1 (Week 1, beginning of semester), T2 (Week 8, mid-semester), and T3 (Week 16, end of semester). At each wave, participants completed the SEWS and SRWSQ in a classroom setting during regular English class periods (approximately 15 min). The timed writing task was administered in a separate session within the same week. The interval between adjacent waves (approximately 8 weeks) was chosen to balance temporal sensitivity with participant retention and to align with instructional cycles. Of the 410 students initially recruited, 368 (89.8%) were retained for analysis; of these, 326 provided complete data at all three waves and 42 had missing data at one or more waves. Attrition analyses comparing completers and non-completers on T1 variables yielded no significant differences (all ps > 0.10).

### 2.4 Data analysis

All analyses were conducted using R (version 4.3.0) with the lavaan package (Rosseel, 2012) and semTools. Given that multivariate normality could not be fully assumed, all models were estimated using the MLR estimator (maximum likelihood with robust standard errors and a Satorra–Bentler scaled chi-square statistic), which is robust to non-normality and non-independence of observations. Of the 368 retained participants, 326 completed all three waves; 42 participants had missing data at one or more waves (11.4% attrition). Primary analyses used full information maximum likelihood (FIML) estimation to retain all 368 participants under the missing-at-random assumption, with gender, age, and T1 writing performance included as auxiliary variables. Completer-only analyses (*n* = 326) yielded substantively identical results and are reported as a sensitivity check. First, confirmatory factor analyses (CFAs) were performed at each wave to evaluate the measurement model. Second, measurement invariance across the three time points was tested sequentially: configural (unconstrained), metric (factor loadings constrained), and scalar (intercepts constrained). Following Chen (2007), invariance was deemed established when the change in CFI (ΔCFI) was ≤0.01, ΔRMSEA was ≤0.015, and ΔSRMR was ≤0.03 (metric) or 0.01 (scalar).

Third, a CLPM with three constructs (L2WSE, SRWS, WP) measured at three time points was estimated. L2WSE and SRWS were modeled as latent variables indicated by their respective subscale composite scores (ideation, conventions, and self-regulation composites for L2WSE; goal planning, idea generation, monitoring, and revising composites for SRWS), and WP was included as an observed variable. The model included autoregressive paths (each variable regressed on itself at the prior wave), cross-lagged paths (each variable regressed on the other variables at the prior wave), and within-wave correlations. Fourth, a RI-CLPM (Hamaker et al., 2015) was estimated as a sensitivity analysis. The RI-CLPM decomposes each observed score into a between-person random intercept and a within-person centered component, thereby isolating within-person dynamics from stable trait variance. Model fit was evaluated using the comparative fit index (CFI), Tucker-Lewis index (TLI), root mean square error of approximation (RMSEA), and standardized root mean square residual (SRMR). Conventional thresholds were applied: CFI and TLI ≥ 0.95, RMSEA ≤0.06, and SRMR ≤0.08 indicated good fit ().

Fifth, to formally test the hypothesized sequential indirect effect of L2WSE on WP through SRWS, bootstrapped indirect effects with 5,000 resamples and bias-corrected 95% confidence intervals were computed. Indirect effects were considered significant when the confidence interval did not include zero. The RI-CLPM was specified following Hamaker et al. (2015): each observed variable was decomposed into a between-person random intercept (with loadings fixed to 1) and a within-person centered component. Within-wave correlations among the within-person components were freely estimated, and cross-lagged paths were freely estimated across the two transitions. Because the design is observational, cross-lagged coefficients from both models are interpreted as model-implied, temporally ordered associations rather than as estimates of causal effects.

## 3 Results

### 3.1 Descriptive statistics and correlations

Descriptive statistics for the three latent constructs at each wave are reported in Table 1. Mean scores for L2WSE, SRWS, and WP all showed a progressive increase from T1 to T3, suggesting general growth over the semester. Skewness values ranged from −0.42 to 0.38 and kurtosis values from −0.61 to 0.72, well within the acceptable range for normality (Kline, 2016). Correlations among the three constructs were moderate to strong within each wave, with L2WSE and SRWS showing the strongest association (*r* = 0.48–0.54 across waves), followed by SRWS and WP (*r* = 0.41–0.48) and L2WSE and WP (*r* = 0.36–0.43). Cross-wave stability correlations were substantial: L2WSE (rT1–T2 = 0.61, rT2–T3 = 0.64), SRWS (rT1–T2 = 0.58, rT2–T3 = 0.60), and WP (rT1–T2 = 0.55, rT2–T3 = 0.57), indicating moderate temporal stability.

**Table 1:** Descriptive statistics and intercorrelations across three waves.

Variable	T1 *M* (SD)	T2 *M* (SD)	T3 *M* (SD)	*r* (T1–T2)	*r* (T2–T3)	*r* (T1–T3)
L2WSE	3.45 (0.58)	3.62 (0.55)	3.78 (0.52)	0.61	0.64	0.55
SRWS	3.28 (0.61)	3.41 (0.59)	3.55 (0.57)	0.58	0.60	0.52
WP	3.92 (0.68)	4.15 (0.65)	4.38 (0.63)	0.55	0.57	0.49

L2WSE = L2 writing self-efficacy; SRWS = self-regulated writing strategies; WP = writing performance; *M* = mean; SD = standard deviation. All correlations significant at *p* < 0.01.

### 3.2 Measurement model and invariance testing

CFAs conducted separately at each wave supported the three-factor measurement model with acceptable fit: T1 (CFI = 0.948, TLI = 0.936, RMSEA = 0.048, SRMR = 0.041), T2 (CFI = 0.953, TLI = 0.942, RMSEA = 0.044, SRMR = 0.038), and T3 (CFI = 0.956, TLI = 0.946, RMSEA = 0.042, SRMR = 0.036). All factor loadings were significant (*p* < 0.001) and ranged from 0.68 to 0.79. Composite reliability (CR) and average variance extracted (AVE) for L2WSE and SRWS at each wave are reported in Table 2; all CR values exceeded 0.80 and all AVE values exceeded 0.50, supporting convergent validity (Fornell and Larcker, 1981). Measurement invariance was then tested across the three waves (Table 3). The configural model fit well (CFI = 0.952, RMSEA = 0.043, SRMR = 0.038). Imposing metric invariance did not significantly deteriorate fit (ΔCFI = 0.004, ΔRMSEA = 0.002, ΔSRMR = 0.004), nor did imposing scalar invariance (ΔCFI = 0.008, ΔRMSEA = 0.001, ΔSRMR = 0.007). All changes were within Chen’s (2007) recommended thresholds, supporting full measurement invariance across time. Internal consistency was satisfactory across all waves: L2WSE (Cronbach’s *α* = 0.89, 0.90, 0.91 at T1, T2, T3, respectively) and SRWS (*α* = 0.88, 0.89, 0.90).

**Table 2:** Composite reliability and convergent validity across three waves.

Construct	Wave	CR	AVE
L2WSE	T1	0.83	0.55
L2WSE	T2	0.85	0.57
L2WSE	T3	0.86	0.58
SRWS	T1	0.84	0.58
SRWS	T2	0.86	0.61
SRWS	T3	0.88	0.63

CR = composite reliability; AVE = average variance extracted, computed from the standardized loadings of the subscale composite indicators at each wave. L2WSE = L2 writing self-efficacy; SRWS = self-regulated writing strategies.

**Table 3:** Measurement invariance tests across three waves.

Model	CFI	TLI	RMSEA	SRMR	ΔCFI	ΔRMSEA	ΔSRMR
Configural	0.952	0.941	0.045	0.038	–	–	–
Metric	0.948	0.940	0.043	0.042	0.004	0.002	0.004
Scalar	0.940	0.933	0.044	0.045	0.008	0.001	0.007

CFI = comparative fit index; TLI = Tucker-Lewis index; RMSEA = root mean square error of approximation; SRMR = standardized root mean square residual. Δ values are relative to the preceding model. All Δ values are within Chen (2007) thresholds.

### 3.3 Cross-lagged panel model

The CLPM demonstrated good fit (CFI = 0.946, TLI = 0.935, RMSEA = 0.046, SRMR = 0.039). Results are summarized in Table 4 and Figure 2. Autoregressive paths were all significant and positive, confirming temporal stability: L2WSE (βT1 → T2 = 0.52, *p* < 0.001; βT2 → T3 = 0.55, *p* < 0.001), SRWS (βT1 → T2 = 0.49, *p* < 0.001; βT2 → T3 = 0.51, *p* < 0.001), and WP (βT1 → T2 = 0.46, *p* < 0.001; βT2 → T3 = 0.48, *p* < 0.001).

**Table 4:** Cross-lagged panel model standardized path coefficients.

Path	*β* (T1 → T2)	SE	*p*	*β* (T2 → T3)	SE	*p*
L2WSE → SRWS	0.21	0.06	<0.001	0.19	0.06	<0.01
SRWS → WP	0.16	0.06	<0.01	0.18	0.06	<0.01
WP → L2WSE	0.17	0.06	<0.01	0.15	0.07	<0.05
L2WSE → WP	0.09	0.05	0.082	0.08	0.05	0.094
WP → SRWS	0.07	0.05	0.115	0.06	0.05	0.143

*β* = standardized path coefficient; SE = standard error. Autoregressive paths: L2WSE *β* = 0.52/0.55, SRWS *β* = 0.49/0.51, WP *β* = 0.46/0.48 (T1 → T2/T2 → T3, all *p* < 0.001). Model fit: CFI = 0.946, TLI = 0.935, RMSEA = 0.046, SRMR = 0.039.

**Figure 2:**
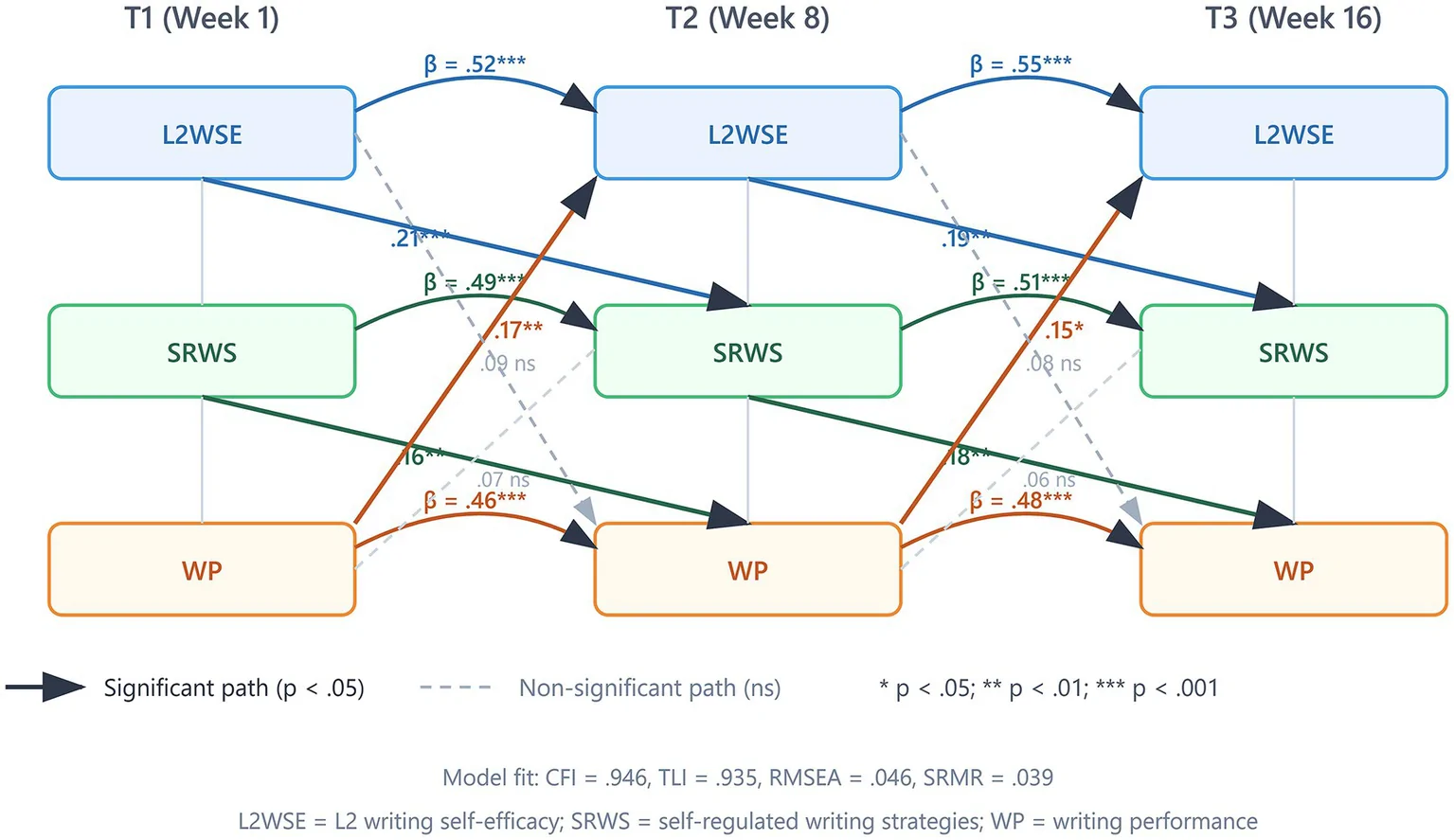
Standardized cross-lagged path coefficients from the CLPM. Solid arrows represent significant paths (*p* < 0.05); dashed arrows represent non-significant paths. Autoregressive stability paths are shown as curved arrows above each construct. Within-wave correlations are depicted as gray vertical lines. Asterisks indicate significance levels: ^*^p < 0.05, ^**^p < 0.01, ^***^p < 0.001. L2WSE = L2 writing self-efficacy; SRWS = self-regulated writing strategies; WP = writing performance. Model fit: CFI = 0.946, TLI = 0.935, RMSEA = 0.046, SRMR = 0.039.

Regarding Hypothesis 1, L2WSE significantly predicted SRWS at both transitions: βT1 → T2 = 0.21 (*p* < 0.001) and βT2 → T3 = 0.19 (*p* < 0.01), indicating that learners with higher self-efficacy reported greater subsequent strategy use. Hypothesis 2 was also supported: SRWS significantly predicted WP at both transitions (βT1 → T2 = 0.16, *p* < 0.01; βT2 → T3 = 0.18, *p* < 0.01), suggesting a prospective association between strategic engagement and subsequent writing improvement. For Hypothesis 3, WP significantly predicted L2WSE at both transitions (βT1 → T2 = 0.17, *p* < 0.01; βT2 → T3 = 0.15, *p* < 0.05), confirming a reciprocal feedback loop from performance to self-efficacy.

Additional cross-lagged paths revealed that L2WSE did not directly predict WP at either transition (βT1 → T2 = 0.09, *p* = 0.082; βT2 → T3 = 0.08, *p* = 0.094), suggesting that the influence of self-efficacy on writing performance is mediated through self-regulated writing strategies rather than acting directly. Similarly, WP did not significantly predict SRWS (βT1 → T2 = 0.07, *p* = 0.115; βT2 → T3 = 0.06, *p* = 0.143), indicating that performance gains do not automatically translate into enhanced strategic engagement.

To formally test the indirect effect of L2WSE on WP through SRWS, we computed bootstrapped indirect effects (5,000 bootstrap samples) for the T1 → T2 and T2 → T3 transitions. The indirect path L2WSE (T1) → SRWS (T2) → WP (T3) was significant (indirect *β* = 0.038, 95% CI [0.012, 0.071]), confirming that self-efficacy influenced writing performance through its effect on self-regulated strategies. The reverse indirect path WP (T1) → L2WSE (T2) → SRWS (T3) was also significant (indirect *β* = 0.036, 95% CI [0.008, 0.065]), supporting the full reciprocal chain. These results provide additional evidence for the sequential and bidirectional dynamics among the three constructs. The magnitude of both indirect effects was small (βs = 0.036–0.038), indicating that while the sequential pathways are statistically reliable, their practical impact over an eight-week interval is modest.

### 3.4 Random-intercept cross-lagged panel model

To disentangle between-person from within-person effects, an RI-CLPM was estimated as a sensitivity analysis (Hamaker et al., 2015; Usami et al., 2019). The model fit well (CFI = 0.938, TLI = 0.924, RMSEA = 0.049, SRMR = 0.042). As shown in Table 5, the within-person cross-lagged effects were directionally consistent with those of the traditional CLPM, although standardized coefficients were slightly attenuated (by 0.01–0.03), consistent with the removal of stable between-person variance. L2WSE predicted SRWS (βT1 → T2 = 0.19, *p* < 0.01; βT2 → T3 = 0.17, *p* < 0.05), SRWS predicted WP (βT1 → T2 = 0.14, *p* < 0.05; βT2 → T3 = 0.16, *p* < 0.05), and WP predicted L2WSE (βT1 → T2 = 0.15, *p* < 0.05; βT2 → T3 = 0.13, *p* < 0.05). The between-person correlations among random intercepts were significant (L2WSE–SRWS *r* = 0.52, *p* < 0.001; SRWS–WP *r* = 0.44, *p* < 0.001; L2WSE–WP *r* = 0.38, *p* < 0.01), indicating that learners who generally had higher self-efficacy also tended to use more strategies and perform better. These results corroborate the temporal dynamics observed in the CLPM.

**Table 5:** Random-intercept cross-lagged panel model within-person effects.

Path	*β* (T1 → T2)	SE	*p*	*β* (T2 → T3)	SE	*p*
L2WSE → SRWS	0.19	0.07	<0.01	0.17	0.07	<0.05
SRWS → WP	0.14	0.07	<0.05	0.16	0.07	<0.05
WP → L2WSE	0.15	0.07	<0.05	0.13	0.07	<0.05

RI-CLPM = random-intercept cross-lagged panel model. Path coefficients represent within-person effects after separating between-person variance. Between-person correlations: L2WSE–SRWS *r* = 0.52 (*p* < 0.001), SRWS–WP *r* = 0.44 (*p* < 0.001), L2WSE–WP *r* = 0.38 (*p* < 0.01). Model fit: CFI = 0.938, TLI = 0.924, RMSEA = 0.049, SRMR = 0.042.

## 4 Discussion

This three-wave longitudinal study investigated the directional and reciprocal relationships among L2 writing self-efficacy, self-regulated writing strategies, and writing performance in Chinese EFL learners. Using both traditional and random-intercept cross-lagged panel models, the findings revealed a coherent temporal chain: self-efficacy beliefs predicted subsequent strategy use, which in turn predicted writing performance, while performance gains prospectively predicted higher subsequent self-efficacy. These results are discussed in relation to theoretical, empirical, and pedagogical implications.

The central theoretical contribution of this study lies in its transition from cross-sectional association to longitudinal directional validation of the motivational–strategic–performance chain in L2 writing. Whereas prior research has documented correlational links among self-efficacy, strategy use, and writing outcomes (Pajares, 2003; Zhou et al., 2022), the present three-wave design with both traditional and random-intercept CLPM provides among the first simultaneous tests of the temporal ordering and reciprocal dynamics among all three constructs in an L2 writing context. This advance moves the field beyond static association toward the modeling of temporally ordered, within-person longitudinal associations.

### 4.1 Theoretical implications

The finding that L2WSE prospectively predicted SRWS provides longitudinal support for a core tenet of Bandura’s (1997) social cognitive theory: self-efficacy beliefs function as motivational drivers of self-regulatory engagement. Learners who held stronger beliefs in their writing capabilities were more likely to deploy goal planning, monitoring, and revision strategies in subsequent weeks. This directional effect is consistent with Zimmerman and Bandura’s (1994) finding that self-regulatory influences and self-efficacy jointly shape writing achievement, and it extends the cross-sectional associations reported by Zhou et al. (2022) in a Chinese EFL context. The direction of this path is also congruent with recent evidence that self-efficacy predicts self-regulated learning strategy use in EFL writing (Teng, 2024) and with latent-profile findings linking efficacy–strategy configurations to writing achievement (Chen et al., 2026).

The absence of a direct path from L2WSE to WP, alongside the significant indirect chain through SRWS, suggests that self-efficacy is associated with performance primarily through its prospective association with strategic action rather than through a direct motivational route. This finding can be situated within the SRL cycle: self-efficacy, as a motivational belief, resides in the forethought phase of Zimmerman’s (2000) model, where it shapes goal setting and task analysis. These forethought processes, in turn, activate the performance phase, in which learners deploy self-control and self-monitoring strategies—the very strategies captured by the SRWS measure. The significant L2WSE → SRWS path thus reflects the transition from forethought to performance phases, while the SRWS→WP path captures the translation of strategic engagement into actual written output. This interpretation aligns with the cognitive process model of writing (Flower and Hayes, 1981; Hayes, 2012), in which motivational resources operate through the strategic processes that constitute actual writing behavior. It also echoes Pajares’s (2003) argument that self-efficacy’s effects on writing are partially mediated by regulatory processes, and it complements the broader self-efficacy–performance literature (Honicke and Broadbent, 2016) by demonstrating this mediational pathway in an L2 writing context.

The reciprocal effect from WP to L2WSE is consistent with the mastery experience pathway described by Bandura (1997): successful writing performance prospectively predicts stronger subsequent self-efficacy beliefs, which in turn predict greater strategic engagement. This feedback loop is consistent with Zimmerman’s (2000) cyclical model, in which self-reflection on performance outcomes feeds back into the forethought phase, shaping subsequent self-efficacy and goal setting. The present study is among the first to demonstrate this reciprocal dynamics in L2 writing using a multi-wave CLPM with both between- and within-person decomposition (cf. Teng et al., 2024).

At the same time, the WP → L2WSE association is open to a competing, bias-based interpretation that deserves explicit consideration. Students who perform well may inflate their subsequent self-perceptions (a self-enhancement tendency) or attribute success to their own ability (a self-serving attributional pattern), and either process could generate a prospective performance-to-efficacy association without any genuine mastery-experience mechanism. Two features of the present results are relevant to weighing these accounts. First, the association remained significant in the RI-CLPM, where stable between-person differences are removed: a purely trait-like tendency to report inflated self-views would be absorbed by the random intercept, so the within-person coefficient indicates that performing above one’s own typical level predicts a subsequent rise in self-efficacy relative to one’s own mean. Second, the effect is prospective rather than concurrent: an immediate self-enhancement reaction to success would be expected to manifest within-wave, whereas the observed association spans an eight-week interval. Nevertheless, the two accounts are not mutually exclusive and cannot be fully separated with the present design, because mastery experience and biased self-perception predict the same observed association. What the within-person RI-CLPM coefficients establish is a temporally ordered, within-person longitudinal association between performance and subsequent self-efficacy; they do not identify the psychological mechanism through which performance information is incorporated into self-beliefs. Designs that directly measure performance attributions or that experimentally manipulate success feedback are needed to distinguish these mechanisms.

The convergence of findings across the traditional CLPM and the RI-CLPM strengthens the internal validity of the present study. The RI-CLPM, which partials out trait-like between-person differences, addresses the critique that traditional CLPM may conflate stable individual characteristics with dynamic within-person processes (Hamaker et al., 2015). The fact that the within-person cross-lagged effects remained significant and in the same direction as those from the traditional CLPM indicates that the observed temporal dynamics are not artifacts of stable trait variance but reflect within-person longitudinal associations estimated net of between-person differences. This finding is particularly important given that the between-person correlations among the random intercepts were substantial, confirming the need to separate the two levels of analysis (Usami et al., 2019).

The bootstrapped indirect effects, although statistically reliable, were small in standardized terms (βs = 0.036–0.038), and their substantive meaning deserves comment. Each coefficient quantifies the change in the outcome, in standard-deviation units, transmitted through the mediating chain over a single eight-week interval; effects of this size are best understood as short-term incremental gains rather than transformative shifts. Several considerations temper a dismissive reading. First, per-interval effects of this magnitude can accumulate across successive instructional cycles, so that sustained reciprocal gains over multiple semesters may be educationally meaningful even when any single-interval effect is small. Second, small lagged coefficients are typical in longitudinal L2 writing research: recent three-wave studies report lagged and indirect effects of comparable magnitude (Dong, 2025; Teng et al., 2024), and the meta-analytic zero-order association between writing self-efficacy and achievement, though medium in size (*r* = 0.29; Sun et al., 2021), aggregates concurrent measurements and therefore cannot be compared directly with conservatively controlled lagged estimates. Third, for classroom practice the results imply that strategy instruction and efficacy-supportive feedback should be expected to yield gradual, cumulative benefits rather than immediate large gains, which argues for sustained implementation across a course of study rather than one-off interventions.

The finding that WP did not significantly predict SRWS warrants further consideration. Although this null effect was not hypothesized, one exploratory explanation is that writing performance gains do not automatically translate into enhanced strategic engagement unless learners are explicitly taught to reflect on and adapt their strategies based on performance feedback. This interpretation aligns with Zimmerman’s (2002) observation that self-regulation is a learned skill that requires deliberate instruction and practice rather than emerging spontaneously from successful performance alone. It also suggests that the self-reflection phase of the SRL cycle may need instructional scaffolding to fully activate the feedback loop from performance to strategy use.

### 4.2 Practical implications

From a pedagogical standpoint, the findings carry direct implications for cultivating both motivational beliefs and strategic competence in L2 writing instruction. Writing instructors are encouraged to design tasks that build learners’ sense of mastery (e.g., by providing process-oriented feedback, scaffolding progressively more complex writing tasks, and celebrating incremental progress), thereby strengthening the self-efficacy–strategy nexus. At the same time, explicit instruction in self-regulatory writing strategies (Graham and Perin, 2007; Teng et al., 2022), including goal setting, idea generation, monitoring, and revising, should be integrated into the curriculum to equip learners with the procedural knowledge necessary to translate motivational resources into writing improvement.

The reciprocal performance–efficacy loop also implies that early writing successes are particularly consequential. Instructors may therefore consider beginning instruction with manageable writing tasks that maximize the likelihood of positive early experiences, consistent with a reciprocal pattern of efficacy, strategy use, and performance over time. Conversely, learners who struggle early may benefit from additional scaffolding to prevent a negative trajectory.

The finding that self-efficacy indirectly influences writing performance through self-regulated strategies highlights the importance of integrated instructional approaches that simultaneously address motivational and strategic dimensions. Writing instructors could implement self-regulated strategy development (SRSD) models, which combine strategy instruction with self-regulation training and have been shown to improve both writing performance and self-efficacy (Graham and Perin, 2007; Rahimi and Fathi, 2022). Teachers can also model metacognitive monitoring during writing tasks, provide structured self-assessment rubrics, and facilitate peer review sessions that reinforce reflective practices. These approaches may help close the gap between performance gains and strategy development by explicitly scaffolding the self-reflection phase of the SRL cycle.

### 4.3 Limitations and future directions

Several limitations should be noted. First, the design is observational: although the three-wave CLPM and RI-CLPM establish temporal ordering, the cross-lagged coefficients are model-implied, temporally ordered associations and do not by themselves establish causal effects, and better model fit does not imply causal correctness. Experimental or quasi-experimental intervention designs are therefore required to validate the directional hypotheses derived from this observational longitudinal study. Second, all data were collected from a single public university in central China, and the sample consisted of undergraduates in their first 3 years of study within one Chinese EFL context; their proficiency levels, instructional exposure, and motivational profiles may differ from those of learners in other institutions, regions, or educational systems, which constrains the external validity of the findings. Future studies should replicate this design across diverse institutions, proficiency levels, and L2 settings. Third, self-efficacy and strategy use were assessed via self-report, which may be subject to response bias; as discussed above, the WP → L2WSE association could partly reflect self-enhancement or attributional processes rather than mastery experience, and designs that measure attributions directly would help to separate these accounts. Incorporating behavioral measures or think-aloud protocols would also enrich the assessment. Fourth, the study spanned a single semester; longer-term dynamics may reveal additional patterns of change, and residual unmeasured time-varying confounders (e.g., instructional changes) cannot be fully ruled out. Finally, the study focused on argumentative writing; future research should examine whether the observed dynamics generalize across genres.

Future research could also extend the model by incorporating additional motivational constructs (e.g., writing anxiety, task value, growth mindset) to capture a fuller range of motivational factors in L2 writing. Multilevel designs that account for classroom- and teacher-level effects would further strengthen causal inferences. Most directly, the directional hypotheses advanced here call for experimental or quasi-experimental verification: a semester-long self-regulated strategy instruction program with classes randomly assigned to intervention and comparison conditions, with self-efficacy, strategy use, and writing performance measured at multiple points, would allow the efficacy–strategy–performance chain to be tested under controlled conditions, and quasi-experimental collaborative or SRSD-based interventions (cf. Rahimi and Fathi, 2022) offer a feasible intermediate step. Until such evidence accumulates, the model-implied, temporally ordered associations reported here should be read as hypothesis-generating rather than as confirmation of causal direction.

## Data Availability

The raw data supporting the conclusions of this article will be made available by the authors, without undue reservation.
